# Supplementary material for: COVID-19 and associations with frailty and multimorbidity: a prospective analysis of UK Biobank participants
Source: Aging Clin Exp Res. 2020 Jul 23;32(9):1897–905. doi: 10.1007/s40520-020-01653-6 (PMC7377312; doi:10.1007/s40520-020-01653-6)
Supplement: Supplementary file 1 — Supplementary file1 (DOCX 20 kb) [file 40520_2020_1653_MOESM1_ESM.docx]

| **Supplementary Table 1**  Comorbidity groupings. |  |
| --- | --- |
| Comorbidity grouping^1^ | Conditions included |
| 1. Painful conditions | Back pain |
|  | Joint pain |
|  | Headaches (not migraine) |
|  | Sciatica |
|  | Plantar fasciitis |
|  | Carpal tunnel syndrome |
|  | Fibromyalgia |
|  | Arthritis |
|  | Shingles |
|  | Disc problem |
|  | Prolapsed disc/slipped disc |
|  | Spine arthritis/spondylitis |
|  | Ankylosing spondylitis |
|  | Back problem |
|  | Osteoarthritis |
|  | Gout |
|  | Cervical spondylosis |
|  | Trigeminal neuralgia |
|  | Disc degeneration |
|  | Trapped nerve/compressed nerve |
| 1. Hypertension | Hypertension |
|  | Essential hypertension |
| 1. Depression | Depression |
|  | Postnatal depression |
| 1. Asthma | Asthma |
| 1. Coronary Heart Disease | Heart attack/MI |
|  | Angina |
| 1. Treated dyspepsia | Gastro-oesophageal reflux (GORD)/gastric reflux |
|  | Oesophagitis /Barrett's oesophagus |
|  | Gastric stomach ulcers |
|  | Gastric erosions/gastritis |
|  | Duodenal ulcer |
|  | Dyspepsia/indigestion |
|  | Hiatus hernia |
|  | Helicobacter pylori |
| 1. Diabetes | Diabetic nephropathy |
|  | Diabetic neuropathy/ulcers |
|  | Diabetes |
|  | Type 1 diabetes |
|  | Type 2 diabetes |
|  | Diabetic eye disease |
| 1. Thyroid disorders | Thyroid problem (not cancer) |
|  | Hyperthyroidism/thyrotoxicosis |
|  | Hypothyroidism/myxoedema |
|  | Graves’ disease |
|  | Thyroid goitre |
|  | Thyroiditis |
| 1. Rheumatoid arthritis, other inflammatory polyarthropathies, systemic connective tissue disorders and systemic autoimmune disorders | Myositis/myopathy |
|  | Systemic Lupus Erythematosus |
|  | Connective tissue disorder |
|  | Sjogren’s syndrome/sicca syndrome |
|  | Dermatopolymyositis |
|  | Scleroderma/systemic sclerosis |
|  | Rheumatoid arthritis |
|  | Psoriatic arthropathy |
|  | Dermatomyositis |
|  | Polymyositis |
|  | Polymyalgia Rheumatica |
|  | Malabsorption/coeliac disease |
| 1. Chronic Obstructive Pulmonary Disease (COPD) | COPD/chronic obstructive airways disease |
|  | Emphysema/chronic bronchitis |
|  | Emphysema |
| 1. Anxiety, other neurotic, stress related and somatoform disorders | Anxiety/panic attacks |
|  | Nervous breakdown |
|  | Post-traumatic stress disorder |
|  | Obsessive compulsive disorder |
|  | Stress |
|  | Insomnia |
|  | Psychological/psychiatric problem |
| 1. Irritable bowel syndrome | Irritable bowel syndrome |
| 1. Alcohol problems | Alcohol dependency |
|  | Alcoholic liver disease/alcoholic cirrhosis |
| 1. Other psychoactive substance abuse | Opioid dependency |
|  | Other substance abuse/dependency |
| 1. Treated constipation | Constipation |
| 1. Stroke and Transient Ischaemic Attack (TIA) | Stroke |
|  | TIA |
|  | Subarachnoid haemorrhage |
|  | Brain haemorrhage |
|  | Ischaemic stroke |
| 1. Chronic kidney disease | Polycystic kidney |
|  | Diabetic nephropathy |
|  | Renal/kidney failure |
|  | Renal failure requiring dialysis |
|  | Renal failure not requiring dialysis |
|  | Kidney nephropathy |
|  | Immunoglobulin A (IgA) nephropathy |
| 1. Diverticular disease of intestine | Diverticular disease/diverticulitis |
| 1. Atrial fibrillation | Atrial fibrillation |
| 1. Peripheral vascular disease | Peripheral vascular disease |
|  | Leg claudication/intermittent claudication |
| 1. Heart failure | Cardiomyopathy |
|  | Hypertrophic cardiomyopathy |
|  | Heart failure/pulmonary oedema |
| 1. Prostate disorders | Prostate problem (not cancer) |
|  | Enlarged prostate |
|  | Benign prostatic hypertrophy |
| 1. Glaucoma | Glaucoma |
| 1. Epilepsy | Epilepsy |
| 1. Dementia | Dementia/Alzheimer/cognitive impairment |
| 1. Schizophrenia (and related non-organic psychosis) and bipolar disorder | Schizophrenia |
|  | Mania/bipolar disorder/manic depression |
| 1. Psoriasis or eczema | Eczema/dermatitis |
|  | Psoriasis |
| 1. Inflammatory bowel disease | Inflammatory bowel disease |
|  | Crohn’s disease |
|  | Ulcerative colitis |
| 1. Migraine | Migraine |
| 1. Chronic sinusitis | Chronic sinusitis |
| 1. Anorexia or bulimia | Anorexia, bulimia/other eating disorder |
| 1. Bronchiectasis | Bronchiectasis |
| 1. Parkinson's disease | Parkinson's disease |
| 1. Multiple sclerosis | Multiple sclerosis |
| 1. Viral Hepatitis | Infective/viral hepatitis |
|  | Hepatitis B |
|  | Hepatitis C |
|  | Hepatitis D |
|  | Hepatitis E |
| 1. Chronic liver disease | Oesophageal varices |
|  | Non infective hepatitis |
|  | Liver failure/cirrhosis |
|  | Primary biliary cirrhosis |
| 1. Osteoporosis | Osteoporosis |
| 1. Chronic fatigue syndrome | Chronic fatigue syndrome |
| 1. Endometriosis | Endometriosis |
| 1. Meniere disease | Meniere disease |
| 1. Pernicious Anaemia | Pernicious anaemia |
| 1. Polycystic ovaries | Polycystic ovaries |
| 1. Cancer | Lifetime diagnosis |
| ^1^ Self-reported lifetime diagnoses by a doctor, during face-to-face interviews at study recruitment (UK Biobank data field 20002), apart from cancer, which was reported during touchscreen questionnaires (UK Biobank data field 2453). Based on previous work by Barnett and colleagues [19] and Nicholl and colleagues [20]. | |
